# Development of HomeSTEAD’s physical activity and screen time physical environment inventory

**DOI:** 10.1186/1479-5868-10-132

**Published:** 2013-12-05

**Authors:** Derek Hales, Amber E Vaughn, Stephanie Mazzucca, Maria J Bryant, Rachel G Tabak, Christina McWilliams, June Stevens, Dianne S Ward

**Affiliations:** 1 Department of Nutrition, Gillings School of Global Public Health, University of North Carolina at Chapel Hill, 245 Rosenau Hall, CB 7461, Chapel Hill, NC 27599-7461, USA; 2 Center for Health Promotion and Disease Prevention, University of North Carolina at Chapel Hill, 1700 Martin L. King Jr. Blvd., CB 7426, Chapel Hill, NC 27599-7426, USA; 3 Clinical Trials Unit (CTRU), University of Leeds, Leeds LS2 9JT, UK; 4 Prevention Research Center in St. Louis, Brown School, Washington University in St. Louis, 621 North Skinker Blvd., St. Louis, MO 63130-4838, USA

**Keywords:** Home environment, Physical characteristics, Physical activity, Screen time, Measurement

## Abstract

**Background:**

The home environment has a significant influence on children’s physical activity, sedentary behavior, dietary intake, and risk for obesity and chronic disease. Our understanding of the most influential factors and how they interact and impact child behavior is limited by current measurement tools, specifically the lack of a comprehensive instrument. HomeSTEAD (the Home Self-administered Tool for Environmental assessment of Activity and Diet) was designed to address this gap. This new tool contains four sections: home physical activity and media equipment inventory, family physical activity and screen time practices, home food inventory, and family food practices. This paper will describe HomeSTEAD’s development and present reliability and validity evidence for the first section.

**Methods:**

The ANGELO framework guided instrument development, and systematic literature reviews helped identify existing items or scales for possible inclusion. Refinement of items was based on expert review and cognitive interviews. Parents of children ages 3–12 years (n = 125) completed the HomeSTEAD survey on three separate occasions over 12–18 days (Time 1, 2, and 3). The Time 1 survey also collected demographic information and parent report of child behaviors. Between Time 1 and 2, staff conducted an in-home observation and measured parent and child BMI. Kappa and intra-class correlations were used to examine reliability (test-retest) and validity (criterion and construct).

**Results:**

Reliability and validity was strong for most items (97% having ICC > 0.60 and 72% having r > 0.50, respectively). Items with lower reliability generally had low variation between people. Lower validity estimates (r < 0.30) were more common for items that assessed usability and accessibility, with observers generally rating usability and accessibility lower than parents. Small to moderate, but meaningful, correlations between physical environment factors and BMI, outside time, and screen time were observed (e.g., amount of child portable play equipment in good condition and easy to access was significantly associated with child BMI: r = -0.23), providing evidence of construct validity.

**Conclusions:**

The HomeSTEAD instrument represents a clear advancement in the measurement of factors in the home environment related to child weight and weight-related behaviors. HomeSTEAD, in its entirety, represents a useful tool for researchers from which they can draw particular scales of greatest interest and highest relevance to their research questions.

## Background

More than 30% of children in the United States (US) are overweight or obese (BMI > 85th percentile) [1]. The high prevalence of overweight and obesity among children is not unique to the US, but is seen in many developed and developing countries as well [2,3]. Excess weight results in numerous physical and mental health problems that arise in childhood and extend out into the adult years [2,4]. Understanding the factors that influence child weight and weight-related behaviors (e.g., physical activity, sedentary behavior, diet) is critical to our efforts to address this challenging obesity epidemic [5]. The environment is recognized to have a large influence on child physical activity and diet, and there is growing recognition of the home and family context, as the most proximal, is perhaps the most influential environment [2,6,7].

Several physical and social factors in the home environment have been shown to influence children’s physical activity, sedentary behavior, and food intake - key behaviors associated with obesity. Ferreira and colleagues’ review identified availability and accessibility of exercise equipment, parental physical activity behaviors and role modeling, logistic support for activity, encouragement for activity, and social norms as having significant associations with child physical activity [8]. Similarly, van der Horst and colleagues’ review identified availability and accessibility of foods, parental dietary intake and role modeling, and controlling or restrictive feeding practices as having significant associations with child diet [9]. Associations between these home environmental factors and child physical activity and diet were at times inconsistent. However, authors of both reviews noted the lack of clear and standardized definitions and instruments used to measure these environmental constructs, which limited the comparison between studies and summarization of findings.

Comprehensive measures of the home’s physical and social environment with solid reliability and validity evidence are critical to understanding how the home influences child physical activity and diet behaviors; however, few such tools exist. Pinard and colleagues’ [10] review of existing measures of the home environment identified 40 instruments, only two of which were deemed comprehensive in nature and provided sufficient evaluation evidence to be useful – the Home Environment Survey [11] (HES) and the Healthy Home Survey (HHS) [12]. However, the respective authors noted several limitations. HES authors suggested additional scale development around sedentary behavior, as these were clearly unique constructs separate from physical activity. HHS authors noted the need for additional items related to the physical activity environment, because the literature in this area was somewhat underdeveloped compared to the diet literature. Additionally, the HHS’s use of an open inventory approach to assess availability and accessibility of foods in the home created challenges with regards to data collection and summarization. While past instrument development has provided a solid foundation, continued advancements in the measurement of the home environment are clearly needed.

The current project represents an effort to advance the measurement of the home physical activity and food environment, specifically in families with children between the ages of 3–12 years old. This paper will describe the development of HomeSTEAD - the Home Self-administered Tool for Environmental assessment of Activity and Diet – which is based on the team’s previous work developing the HHS as well as the rapidly growing literature in this area. This new instrument is designed to provide a comprehensive evaluation of home environmental factors (physical and social) thought to influence children’s physical activity (active and sedentary behaviors) and diet. This new instrument includes four sections: home physical activity and media equipment inventory (physical environment around physical activity), family physical activity and screen time practices (social environment around physical activity), home food inventory (physical environment around food), and family food practices (social environment around food). This paper will describe overall development of this new instrument and methods used to evaluate its psychometric properties. It will also present test-retest reliability and criterion and construct validity evidence of items and scales measuring the physical activity and media equipment inventory. Later papers (under development) will present findings related to the other three sections of the instrument.

## Methods

### HomeSTEAD instrument development

The HomeSTEAD instrument was developed using a mixed methods approach based on a systematic review of the literature, expert review (content validity), and cognitive interviews (face validity).

The Analysis Grid for Environments Linked to Obesity (ANGELO) framework [13] was adopted to help guide identification of relevant constructs. This framework builds off of the social ecological model; it conceptualizes two sizes of environments (micro and macro) each including four types of environments (physical, socio-cultural, political, and economic). Applying this framework to child obesity, the home environment represents a significant microenvironment where physical, social, political, and economic factors interact to influence weight-related behaviors [13,14].

This framework was used to guide the creation of an initial content map identifying constructs within the home environment related to children’s physical activity, screen time, dietary intake, and weight. The content map was then used to guide two systematic reviews of the literature (one around the home physical activity environment, and another around the home food environment [15]. The systematic reviews were used to refine the original content map and identify existing items or scales intended to measure constructs of interest. Items and scales retrieved during this review were cataloged into a database and categorized according to the content map. When existing items were available, the research team reviewed sets of similar items and selected those that were deemed to be the most relevant for that construct. When existing items were not available, the research team developed new items. Where possible, response options were standardized across sections of the HomeSTEAD survey. For example, physical activity-related items generally used 6-point likert-type response scales (e.g., 1 = never, 2 = rarely, 3 = occasionally, 4 = sometimes, 5 = often, 6 = very often; *or* 1 = strongly disagree, 2 = disagree, 3 = slightly disagree, 4 = slightly agree, 5 = agree, 6 = strongly agree).

To assess content validity, the preliminary item pool was distributed to four content area experts for their review. Experts were prompted to provide feedback and suggestions related to content coverage, item relevance and intention, and question format and clarity. Revisions were made based on the feedback; and a 1,007-item self-administered survey was created.

Next, 34 one-on-one guided cognitive interviews were conducted with 27 parents. Participants were recruited through newspaper advertisements, listserv notifications, and community postings. Eligible parents had to have at least one child 3–12 years old with no physical/heath limitations affecting their diet or physical activity, live within 30 miles of the research campus, and be able to speak English. Cognitive interviews were conducted by trained project staff using a structured interview guide. To reduce subject burden, participants were asked to complete only a section of the HomeSTEAD survey: (1) home physical activity, media, and food inventory, (2) family physical activity and screen time practices, or (3) family food practices. However, parents were given the opportunity to participate in a second interview with one of the other sections of the survey. In-home cognitive interviews were conducted for the physical activity, media equipment, and food inventories to assess whether individuals responded to these items by physically checking areas of their homes. Cognitive interviews for the remaining sections were conducted via telephone. During interviews, participants were guided through the survey section item-by-item with prompts from the interviewer to help identify problems with question clarity and comprehension and to better understand how and why respondents selected certain answers. Content analysis of these cognitive interviews occurred in an iterative manner. After completing 4–5 interviews on a section of items, interviewers created a summary report highlighting any issues with individual items. This summary was reviewed and discussed by the project team. Problematic items were revised and underwent additional rounds of cognitive interviews until items were deemed acceptable (no remaining issues regarding clarity or interpretation of items). Across all rounds, there were 9 interviews on the family food practices section, 6 interviews on the family physical activity and screen time practices section, 6 interviews on the home physical activity, media, and food inventories, and 5 interviews on a reduced list of physical activity, screen time, and food practices.

### Physical activity and media equipment inventory

The HomeSTEAD instrument at this stage contained 1017 items divided into four primary sections. The sections include: home physical activity and media equipment inventory, family physical activity and screen time practices, home food inventory, and family food practices. This paper presents data from only the home physical activity and media equipment inventory, which included 304 items and is described in Table 1. Items capture presence, number, accessibility, condition, location, and/or other characteristics of adult physical activity equipment, child fixed play equipment, child portable play equipment, TVs, computers, video games, and portable electronic devices, and yard characteristics. Many items are follow-up questions that can be skipped if certain pieces of equipment are not present in the home.

**Table 1:** Items within HomeSTEAD’s physical activity and media inventory

**Category (# items)**	**Description of items**	**Derived/summary variables**
**Physical activity items**		
Adult exercise equipment (60)	Presence, number, condition, and accessibility of 15 different types of equipment (e.g., cardio equipment, weights, workout DVDs)	Sum of different types of adult exercise equipment present, average condition of equipment, sum of equipment pieces rated as good condition, average accessibility of equipment, sum of equipment pieces rated as easy to access, sum of equipment pieces rated as good condition *and* easy to access
Fixed play equipment (24)	Presence, condition, and accessibility for 8 different types of equipment (e.g., basketball hoop, climbing structure, playhouse)	Total number of different types of fixed play equipment present, average condition of equipment, sum of equipment pieces rated as good condition, average accessibility of equipment, sum of equipment pieces rated as easy to access, sum of equipment pieces rated as good condition *and* easy to access
Child portable play equipment (115)	Presence, number, location, condition, and accessibility for 23 different types of equipment (e.g., balls, push/pull toys, jumping toys)	Total number of pieces of portable play equipment present, average condition of equipment, sum of equipment pieces rated as good condition, average accessibility of equipment, sum of equipment pieces rated as easy to access, sum of equipment pieces rated as good condition *and* easy to access
Yard characteristics (22)	Natural elements checklist (15 items), presence and size of open play space, driveway, perceived sufficiency of yard space and portable equipment, and ownership of and frequency of play with dog	Sum of natural elements present
**Screen time items**		
TVs (8)	Number, as well as location, size, and connection to DVD, recording (e.g., DVR, TiVO), video game, and cable/satellite for up to 6 TVs, and subscription to DVD rental service	Average size of all TVs present, sum of TV connected to cable/satellite
Computers (5)	Number, as well as type (desktop vs. laptop), location, connection to internet, and child access for up to 5 computers	Sum of computers that child is allowed to use
Video game systems (31),	Ownership (yes or no), as well presence, number, location, number of games, and child access for 6 different types of systems	Sum of different types of video game systems present, sum of video games reported, sum of video game systems that child is allowed to use
Portable electronic devices (35)	Presence, number, location, number of games, and child access for 7 different types of devices	Sum of different types of portable electronic devices present, sum of portable electronic device games reported, sum of devices that child is allowed to use, sum of devices located in child’s bedroom
Use of portable screens in the car (4)	Presence of portable DVD or video game devices, frequency of child’s use of the DVD or video games during different length car trips	Average frequency of use of portable DVD or video games in car across all trips, average frequency of use during short car trips and long car trips

### Reliability and validity testing

#### Sample

Recruitment strategies and eligibility criteria similar to those described for cognitive interviews were employed to recruit and screen participants for this reliability and validity testing phase. Demographics were assessed during the screening call and monitored weekly to ensure representation across child age groups (ages 3–5, 6–9, 10–12), income (< or ≥ $50,000), and race (white, black). For example, once sufficient numbers of families with 3–5 year old children were recruited, we stopped enrolling these families and focused on families with older children. If a family reported having more than one child aged 3–12, one child was designed as the reference (the older child was generally selected to ensure recruitment of a sample with a good distribution of age ranges). Consent forms were mailed to participants and collected during the home visit (see below). All methods were reviewed and approved by the University of North Carolina Institutional Review Board.

### Data collection procedures and additional measures

**Figure 1:**
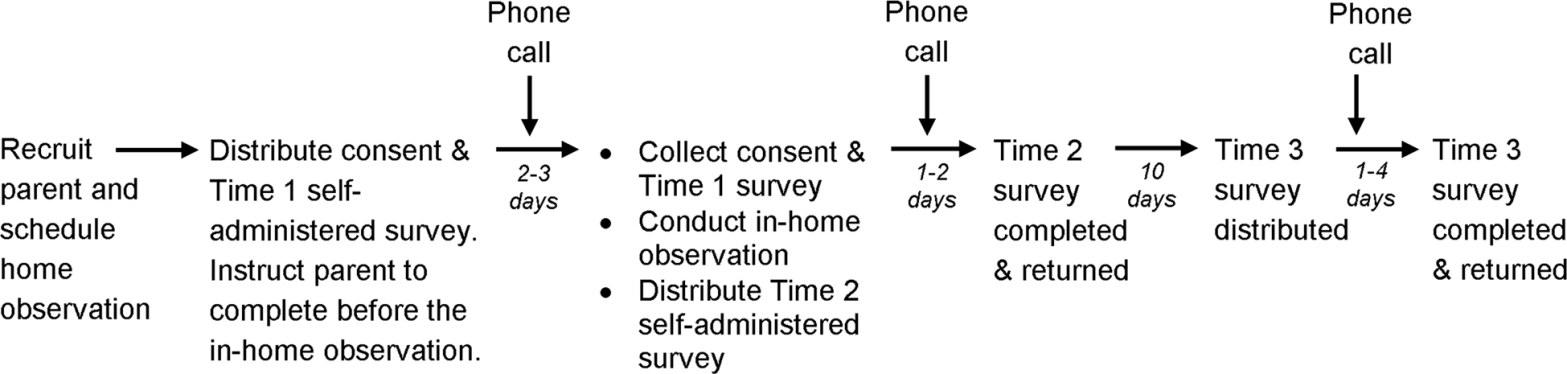
Overview of data collection protocol.

Participants completed all four sections of the self-administered HomeSTEAD survey on three separate occasions over a period of 12 to 18 days and allowed research staff to conduct an in-home observational assessment. The entire protocol was pilot tested with 5 families to ensure feasibility. No problems were encountered during the pilot; therefore, protocols remained unchanged for the rest of data collection. Figure 1 illustrates the steps of this data collection process. Procedures are described in detail below.

Two or three days before the scheduled home-visit, participants were mailed a packet of materials containing: written consent and a paper copy of the HomeSTEAD survey (Time 1). Participants were called the day prior to the home visit to remind them of the scheduled visit and to prompt for completion of the Time 1 HomeSTEAD survey.

Two staff attended each in-home observation. When staff arrived at the home, they collected the signed consent form along with the completed Time 1 HomeSTEAD survey. For the observational assessment, staff requested access to all rooms in the home and inquired about alternative food storage areas (e.g., garage, area outside kitchen). The in-home observational assessment included any items, both physical activity and food, from the HomeSTEAD survey that could be directly observed during the home visit with little or no feedback from the participant. For example, the numbers of TVs, computers, and play equipment were deemed observable, but items asking about value of physical activity or child preferences for active versus sedentary were considered not directly observable within the context of this home visit. At the conclusion of the home visit, staff provided a second copy of the HomeSTEAD survey (Time 2) with instructions to return the survey via mail within 24 hours. Participants were called the following day as a reminder to complete and return the Time 2 HomeSTEAD survey.

Approximately 10 days later, participants received a third copy of the HomeSTEAD survey by mail (Time 3) with instructions to complete and return the survey within 4 days. Participants were called 10–14 days after the home visit as a reminder to complete and return the Time 3 HomeSTEAD survey. If the third survey was not completed and returned within an additional 10 days (even after repeated reminder calls), that participant’s data were not included in the analysis.

#### Additional measures

In addition to the HomeSTEAD survey, parents were asked to complete a short survey about their child’s physical activity and dietary intake (completed along with the Time 1 self-administration). The physical activity items asked parents to report “about how many hours did your child spend” in certain activities (i.e., playing outside, watching TV, and playing video games). The dietary intake items came from a previously validated 19-item instrument and asked parents to report how often their child ate or drank certain beverages and snack foods, [16] with one item added to capture intake of potatoes. Height and weight were also measured on the reference child. Height was measured to the nearest 1/8th inch using a Shorr or Seca stadiometer (Shorr Productions, Olney, MD; Seca Corporation, Columbia, MD); and weight was measured to the nearest 0.1 lb. using a Seca portable electronic scale models 770 and 874 (Seca Corporation, Columbia, MD). Collection of these additional surveys and measures was part of the home visit. Height, weight, sex, and age information were then used to calculate child BMI (weight converted to kilograms divided by height converted to meters squared), which was then used to calculate child BMI percentile using Centers for Disease Control and Prevention growth charts [17].

### Statistical analysis

Derived variables were created by summing or averaging responses to closely related items (see Table 1). For example, data from the 15 different types of adult physical activity equipment were summed or averaged to create variables for types of equipment present, average rating of condition, number in good condition, average rating of accessibility, and number considered easy to access. These were treated as continuous variables. In addition, higher order items were used to create variables with categorical responses. For example, location of each of the TVs reported was used to create dichotomous variables for TV in child’s bedroom and TV in child’s playroom.

To evaluate test-retest reliability, we calculated kappas and the single-measure intraclass correlations (ICC). The single-measure ICC, ICC(1,1) from Shrout and Fleiss [18], is an estimate of reliability given a single random administration. While this is the most appropriate ICC for the intended use of these items and scales, we also calculated the average-measure ICC, reliability if the mean of three scores were used (ICC(1,k)). Both ICC estimates are included in the Additional file 1. The coefficient of variation, standard error of measurement, percent agreement, and number of administrations required to obtain acceptable reliability were also calculated. Using the correlations among responses at the three time points we also assessed reliability of responses to the instrument and stability of the trait [19,20].

To evaluate criterion validity, we calculated kappas, sensitivity, specificity, intraclass correlations and limits of agreement between the observation and the Time 2 self-report. The observational assessment collected by trained research staff served as the “gold standard” for these comparisons. Time 2 survey data were used for these analyses because they were reported closest to the observation. Comparisons between the observation and the results from the Time 1 and Time 3 surveys were also evaluated, but only reported if they differed significantly from the Time 2 results. Construct validity evidence was assessed by examining correlations between the items and scale scores from the HomeSTEAD survey and child BMI percentile or parent-reported eating and physical activity behaviors.

Calculated kappa and ICC scores were compared against established criteria to evaluate strength of the reliability and validity evidence. The work of Landis and Kotch guided evaluation of kappa scores, in which scores of 0.41-0.60 = moderate, 0.61-0.80 = substantial, 0.81-1.00 = almost perfect agreement [21]. ICC scores were evaluated based on criteria proposed by Shrout, in which scores of 0.61-0.80 = moderate and 0.81-1.0 = substantial agreement [22].

## Results

A sample of 129 parents agreed to participate in the study, and 125 (97%) completed the entire protocol (3 self-administrations and 1 home observation). Characteristics of participants are described in Table 2. In brief, parents were predominantly mothers (91%), white (71%) or African American (25%), above median income for area (68% with income ≥ $50,000), and well educated (79% college degree or higher). The average age of the reference children was 7.1 ± 2.9 years old). Participants completed study components (self-administrations and home observation) in a timely manner. On average, there were 3.9 ± 3.7 days between Time 1 and Time 2 self-administrations, 1.8 ± 2.1 days between the home observation and Time 2 self-administration, and 12.4 ± 5.6 days between Time 2 and Time 3 self-administrations.

**Table 2:** Demographics of study participants

	**Percent**
**Parent characteristics**	
Mothers	90.7
Race	
White	71.3
African American	25.4
Other	3.3
Marital status	
Married	79.5
Single	12.3
Income	
<$50,000	32.0
≥$50,000	68.0
Education	
Less than high school	3.3
High school	5.0
Some college	12.4
College degree	42.2
Master’s degree or higher	37.2
**Child characteristics**	
Sex	
Male	51.2
Female	48.8
Age	
3–5 years old	42.6
6–9 years old	35.7
10–12 years old	21.7

### Reliability evidence

Mean scores for continuous variables were very similar across the three parent self-administrations, and single-measure intraclass correlations (ICC1) confirmed good reliability. As seen in Table 3, 97% of the ICC estimates for the primary derived variables were greater than 0.60, only one fell below 0.66. The one low ICC was found for average rating of “condition of portable play equipment” (ICC = 0.24). Across all original and derived variables, the average ICC was 0.68 (range: -0.15 to 1.00), and 72% had ICC estimates greater than 0.60 (additional individual item-level reliability data are provided in Additional file 1). The lowest ICC values (ICC < 0.40) were found for several of the condition ratings for portable play equipment. These items were found to have lower person-to-person variability, with most parents rating the condition of items as “good”.

**Table 3:** Reliability and validity evidence for continuous physical activity environment variables

		**Means**				**Reliability (Time 1, 2, and 3) ICC1**	**Validity (Time 2 vs. OBS)**		
**Label**	**Score**	**Time 1**	**Time 2**	**Time 3**	**OBS**		**N**	**Corr**	**Mean comp (p-value)**
**Adult physical activity equipment**									
Number of types	0-15	5.8	5.8	5.8	4.0	0.94 (0.92, 0.95)	114	0.79	0.000
Condition: Good to Broken	1-4	1.1	1.1	1.10	1.1	0.66 (0.58, 0.74)	99	0.27	0.957
Number in “Good Condition”	0-15	5.1	5.3	5.3	3.7	0.92 (0.90, 0.94)	114	0.79	0.000
Accessibility: Easy to Hard	1-4	1.5	1.4	1.4	1.9	0.67 (0.59, 0.74)	98	0.30	0.000
Number “Easy to Access”	0-15	4.1	4.3	4.1	1.9	0.82 (0.77, 0.86)	114	0.52	0.000
**Fixed play equipment**									
Number of types	0-8	2.1	2.1	2.3	2.0	0.89 (0.86, 0.92)	113	0.87	0.494
Condition: Good to Broken	1-4	1.2	1.2	1.2	1.1	0.84 (0.78, 0.89)	63	0.63	0.352
Number in “Good Condition”	0-8	1.8	1.9	2.0	1.8	0.91 (0.88, 0.93)	114	0.82	0.821
Accessibility: Easy to Hard	1-4	1.4	1.4	1.3	1.2	0.85 (0.80, 0.90)	62	0.84	0.205
Number “Easy to Access”	0-8	1.9	1.9	1.9	1.7	0.92 (0.90, 0.94)	114	0.87	0.559
**Portable play equipment**									
Number of types	0-23	10.9	11.0	11.0	8.9	0.93 (0.91, 0.95)	113	0.81	0.001
Condition: Good to Broken	1-4	1.1	1.1	1.1	1.1	0.24 (0.13, 0.35)	111	-0.03	0.420
Number in “Good Condition”	0-23	9.5	10.0	9.7	8.1	0.86 (0.82, 0.89)	114	0.75	0.001
Accessibility: Easy to Hard	1-4	1.6	1.5	1.5	1.6	0.66 (0.58, 0.74)	108	0.26	0.093
Number “Easy to Access”	0-23	7.1	7.5	7.4	5.3	0.80 (0.74, 0.85)	114	0.58	0.000
**Television**									
Number of TVs in home	Open	2.2	2.3	2.3	2.3	0.97 (0.97, 0.98)	112	0.93	0.935
Average TV size (inches)	24-60	34.4	34.9	35.1	34.3	0.92 (0.89, 0.94)	107	0.75	0.575
Number of TVs with cable	0-6	1.8	1.7	1.8	1.8	0.95 (0.93, 0.96)	104	0.93	0.864
**Video games**									
Number of VG types	0-6	1.0	1.1	1.1	1.1	0.89 (0.86, 0.92)	113	0.76	0.930
Number of VGs	Open	8.0	8.6	9.4	10.7	0.83 (0.78, 0.87)	113	0.70	0.192
Number of VG child is allowed to use	0-6	0.7	0.8	0.8	0.8	0.68 (0.60, 0.75)	114	0.58	0.768
**Computers**									
Number of computers in home	Open	2.2	2.2	2.2	2.2	0.97 (0.96, 0.98)	111	0.81	0.971
Number of computers child is allowed to use	0-5	1.2	1.2	1.2	1.3	0.78 (0.73, 0.84)	114	0.57	0.403
**Portable electronic devices**									
Number of PED types	0-7	1.3	1.4	1.3	1.3	0.85 (0.80, 0.89)	113	0.76	0.733
Number of PEDs	0-63	1.7	1.9	1.8	1.7	0.75 (0.68, 0.81)	113	0.65	0.358
Number of PEDs child is allowed to use	0-7	1.0	0.9	1.0	1.0	0.75 (0.68, 0.81)	114	0.69	0.711
Number of PEDs in child room	0-19	0.4	0.4	0.4	0.3	0.68 (0.59, 0.75)	114	0.45	0.553
**Use of VG in Car:** Never to always									
Average score for all car trips	1-5	1.8	1.9	2.0		0.78 (0.70, 0.85)			
Long car trips (>60 minutes)	1-5	2.0	2.5	2.5		0.76 (0.67, 0.84)			
Short car trips (≤30 minutes)	1-5	1.7	1.5	1.6		0.68 (0.57, 0.78)			
**Use of TV/videos in Car:** Never to always)									
Average score for all car trips	1-5	2.1	2.1	2.3		0.74 (0.66, 0.81)			
Long car trips (>60 minutes)	1-5	2.8	2.9	3.1		0.72 (0.63, 0.80)			
Short car trips (≤30 minutes)	1-5	1.5	1.5	1.5		0.89 (0.85, 0.92)			
**Yard**									
Size of yard (none to large)	0-5	3.8	3.7	3.7	2.8	0.87 (0.83, 0.90)	109	0.31	0.000
Yard space allows for vigorous physical activity (none to a lot)	1-6	4.7	4.8	4.7	4.5	0.80 (0.74, 0.85)	112	0.48	0.064
Amount of portable play equipment in yard (none to a lot)	1-6	2.7	2.7	2.9	2.2	0.72 (0.65, 0.78)	112	0.45	0.010
Number of dogs	Open	0.6	0.6	0.6	0.6	0.79 (0.74, 0.85)	109	0.97	0.999
How often child play outside with dog (never to very often)	1-5	2.5	2.5	2.5		0.88 (0.84, 0.91)			
Number of natural elements	0-15	7.8	8.0	8.2	6.1	0.86 (0.82, 0.90)	113	0.42	0.000

*OBS* Observation.

*Corr* Pearson correlation.

Mean comp (p-values) = P value for the mean comparison.

**Figure 2:**
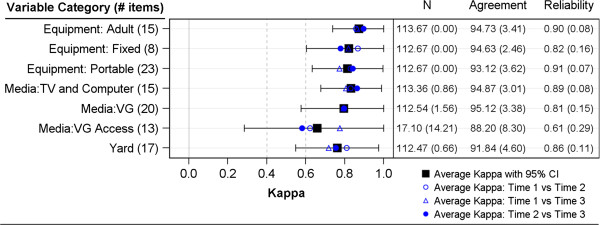
Summary of reliability evidence for categorical physical activity environment variables.

For categorical variables (capturing items presence/absence), percent agreement and kappa reliability estimates generally indicated good reliability. For the major summary variables (Figure 2), percent agreements were above 88% and kappas were generally above 0.70. While still good, parent rating of child access to specific video game consoles was found to have the lowest kappa for this group of variables (average kappa ~0.65). Across all individual items, the average percent agreement was 0.93 (range: 0.72 to 1.00) and the average kappa was 0.80 (range: 0.22 to 1.00).

### Validity evidence

Correlations between Time 2 parent self-administration and the direct observation indicated good agreement for continuous variables, with 73% of our primary derived variables having correlations greater than 0.50 (Table 3). Mean differences and moderate to large deviations were noted for several items related to ratings of access and condition. Only three scores had correlations < 0.40, and a significant mean difference (p < 0.1) between the Time 2 self-report and the observation. One was a rating of yard size, while the other two were related to equipment accessibility. The results show that significant mean differences were found for several equipment and yard items, with project staff consistently observing fewer numbers of items and rating equipment as slightly harder to access compared to parent reports. Across all original and derived variables, average correlation was 0.46 (range: -0.32 to 1.00); however, 50% had correlations greater than 0.50 (additional individual item-level validity evidence can be found in Additional file 1).

**Figure 3:**
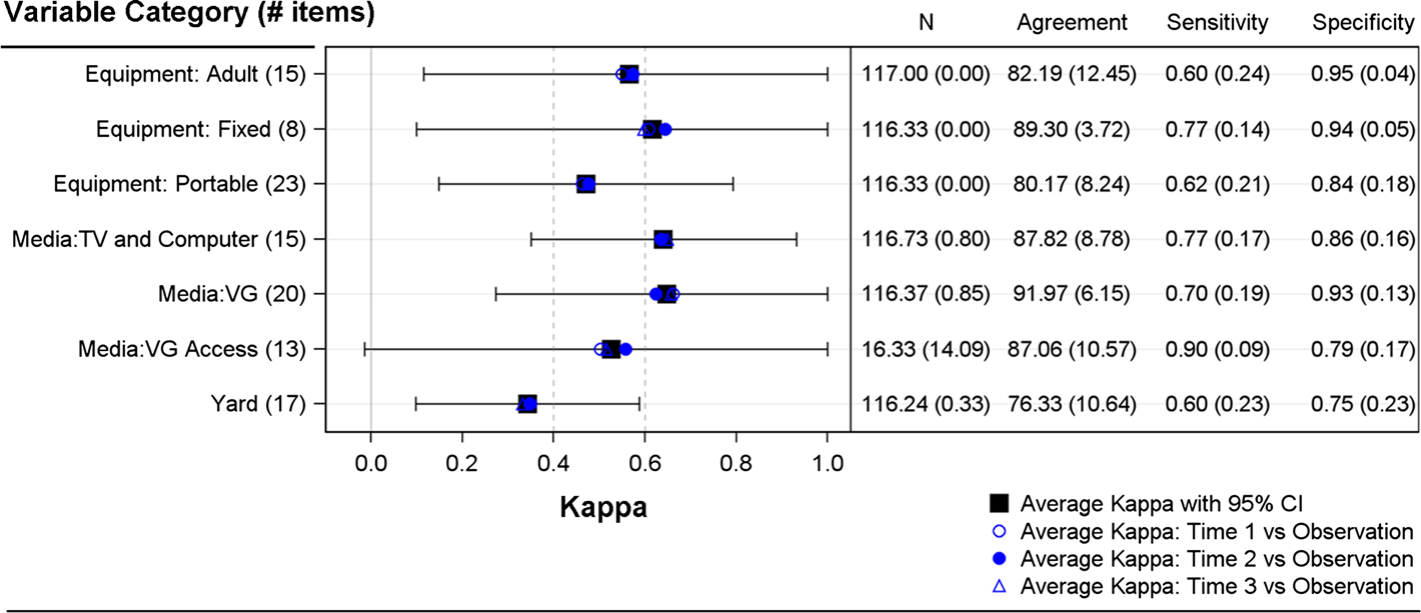
Summary of validity evidence for categorical physical activity environment variables.

For categorical variables, percent agreement and kappa estimates indicated good reliability for the majority of items (Additional file 1). The average percent agreement comparing Time 2 self-administration to the direct observation was 85% (range: 54% to 100%). The average kappa was 0.54 (range: -0.02 to 1.0), with 57% of items having kappa >0.50 and 13% with kappa < 0.30. For these items the true absence (negative) rate, or specificity, was higher (0.86) than the true presence (positive) rate, or sensitivity, 0.69. Figure 3 also shows that kappa estimates were very similar for comparisons of each time point to the observation. This suggests that the time lag between survey administrations affected the validity estimates for these items very little.

Simple correlations between home inventory variables (Time 1 self-administration and observation) and parent reported physical activity of children provides evidence of construct validity (Table 4). Presence of TV and video games were significantly associated with TV and video game time (r = 0.22-0.39). Accessibility of portable play equipment was associated with child outside play time (r = -0.21), and amount and condition of portable play equipment was associated with TV time (r = 0.21-0.26). It is interesting to note that observation was not found to consistently have more, or stronger, correlations compared to the parent report. Significant associations were also observed between child BMI percentile and presence of adult exercise equipment in good condition and easy to access (r = -0.26), and child fixed and portable play equipment in good condition and easy to access (r = -0.25 and -0.23, respectively).

**Table 4:** Correlation of child BMI percentile, outside play time, TV time, video game time, and total screen time with parent reported (time 1) observed (OBS) home environment factors

	**Child BMI percentile**		**Outside play time**		**TV Time**		**Video game time**	
**Label**	**Time 1**	**OBS**	**Time 1**	**OBS**	**Time 1**	**OBS**	**Time 1**	**OBS**
**TVs, VGs and PEDs**								
Number of TVs in house	0.08	0.11	0.04	0.06	0.33^*^	0.35^*^	0.23^*^	0.22^*^
TV in child’s bedroom	0.09	0.16	0.06	0.05	0.31^*^	0.37^*^	0.37^*^	0.24^*^
Number of VG systems	-0.05	0.02	0.01	-0.01	-0.08	0.00	0.22^*^	0.26^*^
Number of VGs	0.04	0.08	-0.01	0.01	-0.05	0.03	0.19	0.29^*^
Number VG systems child is allowed to use	-0.02	-0.01	0.02	0.07	-0.04	-0.02	0.29^*^	0.39^*^
VG in child’s bedroom	0.12	0.09	-0.11	-0.11	0.21^*^	0.07	0.32^*^	0.13
Number of PEDs	-0.07	-0.02	0.04	0.05	-0.08	-0.11	0.13	0.19
Number of PED games	-0.06	-0.04	0.13	0.18	-0.09	-0.07	0.13	0.19
**Adult physical activity equipment**								
Amount of adult physical activity equipment	-0.21	-0.15	0.00	0.03	-0.19	-0.14	-0.10	-0.14
Accessibility of adult physical activity equipment								
(higher score = harder to access)	0.09	0.00	-0.04	-0.05	0.01	0.11	0.08	-0.07
Condition of adult physical activity equipment								
(higher score = broken)	0.19	0.05	0.11	0.04	-0.06	0.27^*^	-0.14	-0.10
Adult equipment in Good condition and Easy to access	-0.26^*^	-0.13	-0.02	0.00	-0.18	-0.17	-0.06	-0.10
**Fixed play equipment**								
Amount of child fixed play equipment	-0.24^*^	-0.14	0.11	0.09	-0.12	-0.14	-0.11	-0.14
Accessibility of child fixed play equipment (higher score = harder to access)	-0.01	-0.10	-0.19	-0.11	-0.05	0.00	-0.04	-0.13
Condition of child fixed play equipment (higher score = broken)	0.14	0.14	-0.06	-0.03	-0.05	0.00	-0.08	-0.06
Child fixed play equipment in Good condition and Easy to access	-0.25^*^	-0.13	0.13	0.09	-0.10	-0.15	-0.08	-0.13
**Portable play equipment**								
Amount of child portable play equipment	-0.18	-0.05	0.02	0.09	-0.24^*^	-0.15	-0.08	-0.06
Accessibility of child portable play equipment (higher score = harder to access)	0.06	0.01	-0.21^*^	0.04	-0.03	0.24^*^	0.02	-0.02
Condition of child portable play equipment (higher score = broken)	0.19	0.05	0.10	-0.02	-0.03	0.26^*^	0.09	0.12
Child portable play equipment in Good condition and Easy to access	-0.23^*^	-0.04	0.05	0.08	-0.19	-0.21^*^	-0.08	-0.05
**Yard**								
Yard size	-0.12	0.09	-0.08	0.09	-0.20^*^	0.13	-0.06	-0.04
Number of natural elements in yard	-0.17	-0.19	0.01	0.03	-0.24^*^	-0.06	0.03	-0.03

*p < 0.05.

*OBS* Observation.

## Discussion

This instrument represents a significant advancement in measurement of the home physical activity and sedentary/screen environment, as well as an important step toward the development of a comprehensive measure of obesogenic factors within the home environment. Few, if any, measures of the home environment have undergone similar systematic and methodical development and rigorous evaluation. Identification of items was based on an articulated theoretical framework with well-defined constructs; it employed a systematic review of the literature to identify potential items; it engaged subject matter experts to ensure content validity; and it incorporated and cognitive interviews to insure item clarity. Evaluation of this new instrument was carefully designed to provide evidence of test-retest reliability, validity of self-report compared to direct observation by trained staff, stability across three self-administrations, and construct validity with child physical activity and screen time. So often one or more of these key elements is lacking [10,21,23], which in turn impacts the quality of the instrument and the confidence in findings based on its use.

This current study demonstrated good reliability and validity evidence for the newly developed physical activity and screen time physical environment inventory within the HomeSTEAD instrument. Results suggest that parent report is consistent and similar to observational data with only one administration of the survey needed to get estimates for most items and scores. In addition, parent report and observation data were similarly related to BMI, outdoor play, and media time bolstering the evidence that parent report is acceptable for collecting this type of household information.

One issue that will need to be addressed in future iterations of the tool is the lack of variation for certain items. Low between subject variation often results in low ICC and Kappa estimates, even when the within person variation is small. In this study, ratings for condition of portable play equipment had a low ICC and basically zero correlation with observation. However, the means, standard error of measurement, and average deviation show that there was very little difference in scores over time, or between parent report and observation (Time 1, Time 2, Time 3, and observation all had a mean score of 1.1 for this item). While parents responded consistently to these items, the similarity in response between participants may limit their usefulness. Before eliminating them, we plan to explore whether better instruction for the response categories or expanding/redefining response options may improve variability. Items should also be evaluated in a more diverse sample. We are also testing options for combining accessibility and condition into a single indicator (e.g., number of items in good condition *and* easily accessible). Preliminary work with this approach suggests that it may produce a better variable than either used alone (Additional file 1).

Agreement between parent report and observation was also a challenge for subjective items assessing factors like condition and accessibility of equipment; however, parent perceptions may be more important than applying some standard criterion when assessing such items. Trained observers tended to rate equipment as being more difficult to access and in poorer condition than parents. While observers provided a more consistent application of standards across homes, perceived accessibility and usability may be as, or more, important within the home environment. If a toy appears to be hard to access to the observer, but the parent consistently makes it available during play time, then the objective rating of accessibility may not indicate how that equipment influences activity. It may be that it is more important to consider perceived access or willingness to access when considering these relationships. In our data, child BMI was more strongly related to parent reported variables than direct observation, supporting the idea that parent report includes an awareness of how they and their children interact with the home environment. This knowledge affects how they respond (e.g., rating an item more accessible because they know it gets used often) and is likely necessary to truly understand the complexity of a child’s physical activity behavior.

While there are existing inventories that assess presence of physical activity and media equipment in the home, the HomeSTEAD’s inventory captures a more complete picture of the home’s physical environment. There are brief checklists such as Rosenberg’s home electronic and physical activity equipment scales [24] that capture the types of electronics in the home (8 items), electronics in child’s bedroom (8 items), portable electronics devices (5 items), and presence and availability of 14 types of physical activity equipment. Sirard’s Physical Activity and Media Inventory [25] represents a more detailed approach that captures the presence of 50 different types of physical activity equipment and 5 types of media equipment, as well as media enhancements like cable/satellite connectivity, number of TV channels available, number of videos or DVDs, number of computer games, type of internet service, and size of TV. Pinard’s recently published Comprehensive Home Environment Survey [26], a 181-item instrument designed to assess the home’s physical and social environment around food, physical activity, and media, includes three scales that assess “physical activity availability” (34 items), “physical activity accessibility” (2 items), and “media availability” (7 items). HomeSTEAD’s home physical activity equipment inventory is, by comparison, more comprehensive than these other measures, capturing presence, accessibility, and condition of adult physical activity equipment (15 types), child play equipment (fixed and portable, 31 types), as well as yard characteristics. Similarly, its media equipment inventory is more detailed, capturing presence, location, accessibility, and connectivity for a variety of screens and media (15 types). As the construct validity evidence suggests, this more comprehensive data may be important in understanding how the home environment is influencing child behavior.

Tests of construct validity showed some significant associations between child screen/media behavior and the home environment in the expected direction. Analyses in this study showed that presence of TV and video games were associated with children’s TV time, video game time, and screen time (r = 0.22-0.41, indicating low to moderate associations). The number of TVs in the home and presence of a TV in the child’s bedroom were significantly associated with TV time, video game time, and total screen time. These associations were generally consistent regardless of whether data were based on parent report or observation. Associations between the number of TVs in the home and/or presence of TV in child’s bedroom and TV viewing behavior have been observed consistently across samples of children of difference ages, races/ethnicities, SES groups, and countries [27-31]; however, there are a few exceptions showing null or inconsistent results [32-34]. Our data did not show an association between TV presence in the child’s bedroom and child BMI percentile, although there is some evidence in the literature of an association [29,35,36]. The number of video game systems in the home was significantly associated with video game time, but not TV time or total screen time (except when the video game is in child’s bedroom). While other studies have accessed presence of video games and video game use [27,30], these items are often merged into larger screen time variables and results do not look for this specific association.

Associations were also observed between variables related to portable play equipment and child outside play time, TV time, and total screen time. Amount, accessibility, and condition of equipment all appear to be associated with child activity, particularly TV and screen time. Amount of portable play equipment (reported by parents) was inversely associated with TV time and total screen time; less accessible equipment was associated with more TV time and total screen time, as well as less outside time; and poorer condition of equipment was associated with more TV time and total screen time. The analysis in the present study is unique from most others in that it looked at associations between portable play equipment and outside time, but also TV, video game, and total screen time. Most studies look for possible associations between portable play equipment and moderate activity, vigorous activity, and/or weight. Trost and colleagues found no association between home physical activity equipment and child moderate or vigorous activity [37]. Similarly, Byun and colleagues were unable to detect an association between physical activity equipment and sedentary behavior [38]. Crawford and colleagues explored associations with physical activity equipment and child weight, but again were not able to detect significant associations [35]. The findings in this study may suggest the importance of measuring not just presence of the equipment, but additional factors such as accessibility and condition of that equipment.

A comprehensive tool that is able to assess the home’s physical and social environment around physical activity and nutrition holds great potential for advancing our understanding of the home environment’s impact on children’s weight-related behaviors. The goal of the HomeSTEAD project is to create one instrument with multiple scales that provides a comprehensive picture of the home environment. The entire instrument may appear long (with 1,017 items); however, it can generally be completed in an hour (based on parent-reported estimates collected during the pilot phase). The reason for this is that it was designed to capture a lot of information but with minimal burden from the participant. For example, in the physical activity inventory, participants skip many of the sub-items, because they only need to provide information about equipment they have in their home. The instrument asks about more than 60 different types of equipment, but participants generally have no more than a third of these in their home. The HomeSTEAD instrument is also intended to be flexible to meet the needs of other researchers. We purposefully provide reliability and validity evidence for all scales within the instrument as well as individual items so that future studies have the option of using the entire instrument, select scales, or individual items, depending on their research questions. We encourage the use of HomeSTEAD by other researchers, because the use of common scales and items would greatly enhance comparability across studies. Being able to compare more directly across studies has the potential to enhance the speed at which researchers can understand relationships between home environment characteristics and child physical activity and nutrition behaviors.

While this study had many strengths, limitations are unavoidable. The primary limitation was the homogeneity of sample, which was primarily white (71%) and well-educated (79% with an undergraduate degree or higher). The instrument would benefit from additional testing in samples with more racial and ethnic minorities and low-income families. Preliminary evidence suggests that reliability and validity evidence in specific subgroups (i.e. boys vs. girls, preschool vs. elementary school age, and income < 50,000 vs. 50000+) are very similar with few meaningful differences (data not presented). A second limitation of the current study is the use of brief parent-report screeners to assess child behaviors around diet and physical activity. The instrument would benefit from additional testing to explore the relationship between items and an objective monitor of physical activity. These are critical next steps in the development of this comprehensive measure which will greatly inform item retention.

## Conclusions

The development of the HomeSTEAD instrument represents a clear advancement in the measurement of the factors within the home environment related to child weight and weight-related behaviors. HomeSTEAD’s home physical activity and media equipment inventory demonstrated strong reliability and validity evidence; the few exceptions were related to items capturing the condition of equipment. Results were stable across administrations. While this paper presents evidence on only one of the four sections of the instrument, the HomeSTEAD survey in its entirety provides a comprehensive measure from which researchers can pull particular scales of greatest interest and highest relevance to their research questions.

## Abbreviations

ANGELO: Analysis grid for environments linked to obesity; BMI: Body mass index; HES: Home environment survey; HHS: Healthy home survey; HomeSTEAD: Home self-administered tool for environmental assessment of activity and diet; ICC: Intraclass correlation coefficient; PED: Portable electronic device; SES: Socio-economic status; US: United States; VG: Video game.

## Competing interests

The authors declare that they have no competing interests.

## Authors’ contributions

DW served as the Principal Investigator for the HomeSTEAD project, with DH, MB, and JS serving as co-investigators. DH, AV, RT, and CM participated in reviewing the literature and retrieval and creation of items. CM oversaw cognitive interviews and summarization of interpretability issues, incorporating feedback from DH, AV, RT, and DW to improve item clarity. SM and CM oversaw collection of reliability and validity data. DH conducted analyses. DH, AV, SM, MB, RT, CM, JS, and DW participated in data review and interpretation of analyses. DH and AV led manuscript development, with contributions, edits, and review by SM, MB, RT, CM, JS, and DW. All authors read and approved the final manuscript.

## Supplementary Material

Additional file 1 Item-level reliability and validity estimates. Click here for file
